# Expert interviewers’ approach to navigating forensic interviews with adolescents who are reluctant to disclose sexual abuse

**DOI:** 10.1080/13218719.2024.2362134

**Published:** 2024-09-03

**Authors:** Dirkje D. Gerryts, Sarah L. Deck, Martine B. Powell

**Affiliations:** Centre for Investigative Interviewing, Griffith Criminology Institute, Griffith University, Brisbane, QLD, Australia

**Keywords:** adolescent victims, investigative interviewing, reluctance, support

## Abstract

Adolescents are often reluctant to disclose experiences of sexual abuse in forensic interviews. In these situations, there is consensus that interviewers should respond supportively, yet they appear to have considerable difficulty doing so. In the current study, we sought to provide practical guidance on how interviewers can adopt a supportive approach when interviewees are reluctant. Twenty-one expert interviewers were asked how they engage and support adolescents who are reluctant to share information when sexual abuse is suspected. The findings indicate that the expert interviewers approach reluctance by leaning into and exploring the interviewee’s perspective, and tailor their response based on an awareness of the unique needs of the interviewee. Several key support strategies were identified to facilitate this approach. The findings of this study provide practical guidance for how interviewers can approach reluctant interviewees, while also generating novel directions for future research.

Interviewers are often faced with the challenge of gathering sensitive and personal information from reluctant adolescent interviewees in investigative interviews. Many adolescents are reluctant to share information when forensically interviewed (Augusti & Myhre, 2021; Dianiska et al., 2023; Hershkowitz et al., 2014; McElvaney & Culhane, 2017). This is the case for a variety of reasons, including having a greater awareness of the consequences of disclosure and a desire to protect the offender (Giroux et al., 2018; Holder et al., 2023; McElvaney et al., 2022; see also Lemaigre et al., 2017, for review). To promote engagement with adolescent interviewees, and to facilitate their comfort, it is paramount that interviewers are equipped with the skills to respond effectively when interviewees appear reluctant. There is evidence, however, that interviewers have difficulty responding appropriately in these moments (Ahern et al., 2014; Hershkowitz et al., 2006). This shortfall in interviewer practice may arise due to a lack of awareness on how to approach these situations on a practical level. In the current study, we aimed to provide practical guidance for how interviewers can respond effectively when interviewees are reluctant. To do so, we explored the perspectives of interviewers, identified as experts in interviewing adolescents, on how they approached conversations with reluctant interviewees, as these professionals are likely to have valuable experiential insight into this subject.

Reluctance refers to verbal or non-verbal behaviours that reflect direct or indirect unwillingness to respond to an interviewer’s questions (Lewy et al., 2015). There is an array of potential behavioural indicators of reluctance, including overt refusals to share information, expressions of heightened emotions such as crying, and other behavioural indicators that are more subtle and more difficult to detect, such as changing the conversational topic (e.g. Henderson et al., 2021). When behaviours such as these arise, the importance of interviewers responding appropriately is threefold. First, reluctant interviewees provide less forensically relevant information (Ahern et al., 2018; Blasbalg et al., 2018; Hershkowitz et al., 2006), which limits the evidence available to lay charges and impacts the perceived credibility of the interviewee and their report (Martschuk et al., 2022). Ultimately, these factors undermine the opportunity for the case to progress to court and secure a conviction (Henderson et al., 2021; Martschuk et al., 2022). Second, overlooking reluctant presentations places the wellbeing of the interviewee at further risk of harm. Interviewers who continue to press for information concerning the allegation despite the presence of reluctance are viewed as threatening (Hershkowitz et al., 2006). In addition to compromising the experience of the interviewee, feelings of discomfort and resentment resulting from inappropriate responses to reluctance can also limit the likelihood the interviewee will remain engaged in the case and subsequent trial involvement (Ahern et al., 2018; Henderson et al., 2021; Katz et al., 2012). Third, interviewees who do not disclose abuse may not be directed to appropriate support services and therapeutic interventions (Theimer et al., 2020), which can enhance the risk of ongoing mental health concerns and maladaptive coping behaviours (Dye, 2018; Gruhn & Compas, 2020; Spalletta et al., 2020). Given the implications of responding inappropriately to interviewee reluctance, it is important to better understand how interviewers can best support interviewees in these circumstances.

There is consensus that interviewee reluctance should be addressed through the provision of non-contingent support (e.g. Blasbalg et al., 2019; Lamb et al., 2018), and various socio-emotional support elements have thus been embedded in most forensic interview protocols (e.g. see, revised National Institute of Child Health and Human Development, NICHD, protocol, Lamb et al., 2018). One interview component that has long been recognised as important, and that provides socio-emotional support, is the inclusion of a rapport-building phase (i.e. ‘narrative practice’ with children), which involves discussing an innocuous topic prior to the alleged abuse. This phase of the interview is known to yield numerous cognitive and social benefits (Powell & Brubacher, 2020; Roberts et al., 2011). Some of these benefits include an opportunity for the child to practise responding to the interviewers’ questions (Anderson et al., 2014; Whiting & Price, 2017), while also promoting the interviewees’ willingness to share information, because it helps interviewees become more comfortable and familiar with the interviewer (Abbe & Brandon, 2013; see, Roberts et al., 2011, for a review).

In addition to establishing some rapport prior to the interview, researchers and practitioners have more recently identified the need to better address the emotional needs of the interviewee *throughout* the interview process (Ahern et al., 2014; Hershkowitz et al., 2017; Lamb et al., 2018). Such an awareness has prompted revisions to various interview protocols, of which the most widely researched is the revised NICHD protocol (Lamb et al**.,** 2018). The revised NICHD protocol includes 26 supportive techniques and utterances, alongside example statements of support, derived from six overarching categories (e.g. addressing the child in a personal way, reinforcement). These supportive strategies include expressions of support during the rapport-building phase (e.g. express interest in the child’s experiences, such as ‘I really want to know you better’), as well as various general expressions of support (e.g. positive reinforcement of the child’s efforts, for instance ‘thank you for sharing that with me’). Some recommended expressions of support may additionally be useful for interviewers to adopt specifically in response to expressions of reluctance (e.g. encouraging disclosure; ‘It’s really important that you tell me if something happened to you’), although as we consider in greater detail below, there is a need for more in-depth guidance on how interviewers can navigate interactions with interviewees who are reluctant, beyond the provision of isolated support statements.

The use of non-suggestive supportive techniques and utterances, including those recommended in the revised NICHD protocol, have been the subject of extensive evaluation and research. Findings from both field and laboratory settings suggest that in general, verbal supportive techniques and utterances reduce reluctance and encourage interviewee participation in forensic interviews, and thus enhance the amount of forensically relevant information obtained (Ahern et al., 2014, 2018; Blasbalg et al., 2018; Hershkowitz et al., 2006; Karni-Visel et al., 2019; Teo & Lamb, 2013). One study, for example, analysed the interviews of 254 children (age 4–14 years) who were expected to be highly reluctant, having disclosed multiple incidents of physical abuse by their parents (Blasbalg et al., 2019). Children were interviewed using either the standard NICHD protocol (which does not include a focus on the provision of socio-emotional support) or the revised NICHD protocol (which includes a focus on the provision of socio-emotional support techniques and utterances). Children interviewed using the revised NICHD protocol were both less reluctant and more informative than those who were interviewed using the standard protocol, illustrating the effectiveness of general support strategies in ameliorating interviewee reluctance.

Despite robust evidence for the inclusion of supportive techniques and utterances in interview protocols, interviewers do not appear to be responding appropriately in moments that interviewees appear reluctant. Indeed, the evidence reveals that in the absence of nuanced guidance surrounding the provision of support in response to interviewee reluctance, interviewers tend to adopt an *unsupportive* approach, using questioning styles that include intrusive and repetitive questioning, confronting the child and increased interviewer talkativeness (Hershkowitz et al., 2006; Lewy et al., 2015; Teoh & Lamb, 2013). Although there is evidence that the revised NICHD protocol (and the enhanced focus on socio-emotional support) enhances the use of supportive utterances in general (Ahern et al., 2014; Blasbalg et al., 2019; Hershkowitz et al., 2015), this does not appear to be the case specifically in response to interviewee reluctance. For instance, when 199 forensic interviews with children (aged 3–13 years) were analysed, interviewers using the revised NICHD protocol uttered more supportive statements than those using the standard protocol (Ahern et al., 2014). Yet in response to interviewee reluctance, all interviewers, including those using the revised NICHD protocol, failed to provide adequate support – responding similarly to reluctant and non-reluctant interviewee behaviours. It is because of these findings that experts recommend interviewers respond supportively when interviewees appear reluctant, rather than continuing on with allegation-related questioning (e.g. Hershkowitz et al., 2017; Katz et al., 2012). How to address interviewee reluctance in a practical and more nuanced way, however, is unclear.

One research method that can provide rich insight into how to respond to reluctance on a practical level is to learn from the perspective of interviewers who have extensive experience navigating interviewee reluctance. In the current study, we adopted this approach using thematic analysis. Specifically, a cohort of interviewers, identified as experts in interviewing adolescents, were asked how they navigate conversations about alleged sexual assault with reluctant interviewees. These responses were analysed to identify commonalities in how the expert interviewers effectively engage with, and support, reluctant interviewees.

## Method

### Participants

Investigative interviewers with expertise in interviewing adolescent complainants of sexual assault were purposively sampled. Potential interviewers were identified from specialised interviewing centres based in different regions of the United States. To identify interviewers, a senior supervisor from these specialist interviewing centres, with responsibilities across all sites, nominated individuals for inclusion in the study based on three criteria: (a) a minimum of 5 years interviewing experience, particularly with adolescents (aged 12–18 years); (b) had completed training in best-practice interviewing with ongoing supervision; and (c) had conducted interviews that had been observed by the nominating senior supervisor. Individuals who met these criteria were invited to participate, and recruitment continued until no new themes emerged (Sim & Wright, 2000).

The final sample consisted of 21 expert interviewers (*n* = 21, *n* = 20 females) who had an average of 14.35 years of interviewing experience (range = 5–30 years, *SD* = 7.47). The interviewers conducted an average of 16.57 interviews a month (range = 5–67, *SD* = 15.04), of which 10.38 (range = 2–40, *SD* = 10.16) were with adolescents. Interviewers who conducted fewer interviews were typically in a supervisory or management role, which involved supporting other interviewers. The interviewers had completed forensic interviewing training in a diverse range of protocols. In total, 18 different forms of training had been completed across the interviewers, and the most common were National Child Advocacy Centre training (*n* = 16), Child-First Interviewing (*n* = 8) and the American Professional Society on the Abuse of Children (*n* = 6). All interviewers had also attained degrees in higher education, which were predominantly in the social sciences. Participants’ highest qualifications were a Bachelor degree (*n* = 6), Master’s degree (*n* = 14) and a Doctor of Philosophy (*n* = 1). Ethics approval was granted by the University’s Human Research Ethics Committee.

### Materials and procedure

After being provided with an overview of the aims of the research, nominated interviewers consented to involvement by returning a signed consent form. Interviewers provided basic demographic information on the consent form, including years of experience interviewing children and the average number of interviews they conducted each month. The research interviews were conducted by one of two researchers (both study authors) and were conducted with each interviewer individually. During the research interview, the interviewers were asked about three main topics: (a) their experiences and impressions of interviewing adolescent complaints of alleged sexual assault, (b) strategies they use to engage and support reluctant adolescent interviewees, and (c) other general support strategies they use when approaching challenging interviews with adolescents. The interviews followed a semi-structured interview format to allow the researcher to flexibly follow up on topics raised by the interviewer.

The research interviews were conducted over an online platform and were, on average, 59.90 min in duration (range = 46–77 minutes, *SD* = 8.36). All interviews were audio recorded and transcribed verbatim, and potentially identifying information was removed prior to analysis.

### Data analysis

The interviewers’ responses were analysed through a grounded theory approach, which involved analysing responses inductively, rather than deductively from an existing theory or framework (Glaser & Strauss, 1998). Our approach to analysis was also collaborative in nature given the value of interpretive discussion held between multiple coders in qualitative research (compared quantitative designs wherein multiple coders are necessary to establish coding consistency and thus reliability; Barbour, 2001).

Interview transcripts were initially read by all study authors. The authors discussed the transcripts and noted common themes. Next, the first two authors subjected each transcript to open coding, which involved a line-by-line analysis of statements and concepts within statements to identify emerging themes. The interviewers’ responses were organised into categories themes to reduce the data to meaningful units of analyses and to identify emerging themes (Miles & Huberman, 1984). The two researchers engaged in collaborative discussion at multiple points throughout this process, and any discrepancies in interpretation were discussed and resolved. The final list of themes was agreed on by all researchers. A considerable volume of themes emerged from the analysis. In this study, we describe themes connected to how the professionals approach conversations with adolescents who appear reluctant; themes not relevant to this focus are not considered further. To ground the results in the data quotes have been included in the findings below, and these have been edited to correct grammar and improve readability.

## Results

Two broad themes emerged from the analysis. The first describes an underlying framework that guided how the interviewers applied supportive strategies, and the second addresses specific support strategies the interviewers used to facilitate this approach.

### Guiding framework for the application of supportive strategies

The first theme addresses underlying principles that guided the interviewers’ approach to applying support when interviewees are reluctant. Four subthemes were evident, and these are summarised in Table 1: **(**a) the necessity of a foundation of best-practice questioning, (b) effective responses to reluctance stem from a basis of rapport, (c) respond appropriately to specific moments of reluctance, and (d) terminate the interview when necessary.

**Table 1.** Guiding framework for the application of supportive strategies.

Subtheme	Key considerations
The necessity of a foundation of best-practice questioning	The interview conversation, including responses to reluctance, should be underpinned by the flexible use of best-practice questioning principles.
Effective responses to reluctance stem from a basis of rapport	Effective responses to reluctance cannot be considered in isolation from the foundation of rapport that has been established with the interviewee up until that moment of reluctance.
Respond appropriately to specific moments of reluctance	Responses to reluctance stem from an understanding of the interviewee and the specific reason(s) for their reluctance in a given moment.
Terminate the interview when necessary	When supportive techniques do not facilitate engagement with the interviewee, it may be appropriate to end the interview.

#### The necessity of a foundation of best-practice questioning

The language of the interview, including how the interviewers navigated reluctance, was underpinned by best-practice questioning principles. The interviewers were aware of the need to use open-ended questions, and used focused questions to explore and respond to reluctance to the extent that it was necessary. Focused questions were carefully chosen to empower the interviewee to express their perspective. The interviewers were also aware of expressions of support that were appropriate in their role as a neutral information gatherer. For example, they would only comment on observable behaviours (e.g. ‘I see tears, tell me about your tears’) and were mindful not to make inferences about how the interviewee was feeling. It was apparent that the interviewers, who were highly trained in best-practice interviewing, were able to apply these principles flexibly to respond to specific moments of interviewee reluctance.

When you have experience doing forensic interviews for a long time, you can be more flexible. You’re more comfortable with the language and with the interview structure, which allows you to be more flexible when you have that resistant teen, and for them to be more of themselves in the process. But when you’re a new interviewer, you’re in your head too much – you’re thinking, for example, What’s my next question? How do I make that an open question? But now I’m able to really tune into trying to understand the kid that I have in front of me. What are they showing me about who they are? What’s the barrier that I’m hearing? And how can I bring those pieces of information out? I’m always looking for the nuggets of information that the kid tells me. Maybe it’s one little piece that they talked about like, ‘I didn’t even want to come in the car today’. So then I’ll say, ‘you said you didn’t even want to come in the car today, what was that about?’ and then, ‘but you came here, so tell me about that?’ You can use those nuggets of information that they share and explore it further. (P03, master’s degree, 9 years’ experience)

#### Effective responses to reluctance stem from a basis of rapport

The data highlighted that the effectiveness of responses to specific moments of reluctance depend (in part) on the level of rapport that has been built with the interviewee. The interviewers perceived effective rapport to be based on a connection that is fostered throughout the entire interview process. The interviewers noted that this rapport is built in a variety of ways – for instance, by the interviewer’s attentiveness to the interviewee throughout the interview (including on non-offence-related topics), and the extent to which the interviewer had provided appropriate support in previous moments in the interview. The interviewers considered that when moments of reluctance arise, the degree to which the interviewer had previously developed rapport with the interviewee influenced the interviewee’s willingness to engage in a dialogue about that reluctance. Thus, it was clear that expressions of responsive support cannot be considered in isolation from the degree to which interviewers had developed rapport with the interviewee up until that point in the interview.

There’s a lot of science about what kind of questions to ask, but when we are talking about addressing reluctance, it really comes back to being responsive and showing support. Connecting with them and having rapport is a job of the interviewer throughout the whole interview, not just a phase in the first five minutes. (P19, master’s degree, 20 years’ experience)

#### Responding appropriately to specific moment of reluctance

When reluctance arises to the extent that it impacts the interviewees’ capacity to engage in the interview, the interviewers noted that they acknowledge and respond to that reluctance. Their approach in doing so is centred around the unique needs of the interviewee before them. That is, the interviewer’s attentiveness to the interviewee, coupled with an assessment of what type of support strategy would be most appropriate, determines how they respond to specific moments of interviewee reluctance. The interviewers reflected that the way that they evaluate the most appropriate support strategies is based on what they understand about the interviewee, including the specific factors(s) underlying their reluctance. Thus, it was clear that the interviewers’ responses to moments of reluctance hinged from an assessment of the appropriateness of that response, for that interviewee, in that specific moment of reluctance.

I try to read the room and read the kid I’m working with before I respond to their reluctance. A lot of it is clinical judgement. I’m asking myself ‘Do I think this child would be receptive to this approach?’. (P16, master’s degree, 21 years’ experience)

#### Terminating the interview when necessary

Finally, it is important to note that while the interviewers spoke of how they navigate reluctance and use a variety of supportive strategies to do so, it was clear that if they cannot facilitate some engagement and dialogue with the interviewee, they reassess the situation and consider ending the interview.

### The application of various support strategies

The interviewers applied various supportive strategies to facilitate the approach to navigating interviewee reluctance as explained above. Three subthemes emerged: recognise and identify reluctance, explore reluctance, and respond to reluctance. For each of these subthemes, specific support strategies were recommended. These subthemes and strategies are described in Table 2.

**Table 2.** Thematic framework describing the application of various support strategies, including associated support strategies and example supportive utterances.

Subtheme	Support strategies	Example support statements and prompts
Recognise and identify reluctance	Pre-interview preparation	
	Narrative practice	*Tell me what you like to do for fun?*
Explore reluctance	Explore feelings	*How do you feel about being here today?* *How are you feeling right now?*
	Explore thoughts	*Tell me what you’re thinking about right now?* *Do you have any worries about talking to me today?*
	Explore topics that the interviewee is willing to share	*What can we talk about in here today?* *Is there something that you don’t want to talk about? Tell me about not wanting to talk about it.*
Respond to reluctance	Address reluctance	
	Enhance interview transparency	*There are some people watching us talk today and before you and I finish talking, I’ll step out and speak to them.*
	Provide reassurance	
	Autonomy	*If you want to talk, that’s okay, if you don’t want to talk, that’s okay as well.*
	Privacy	*If we talk about something today and you don’t want me to tell your mom, that’s fine, just let me know.*
	Pacing	*You can take as much time as you need. I’m not in any hurry, I’m not in any rush.*
	Feelings	*You can feel however you want to feel.*
	Normalise the conversation	*I’ve talked to lots of boys your age.*
	Accommodate reluctance	
	Redirect the line of questioning	*You told me earlier that you have a dog. Tell me more about your dog.*
	Offer the opportunity to write it down	*Is there something that would be easier for you to write instead of saying out loud?*

#### Recognise and identify reluctance

First, the analysis pointed to the perceived value of recognising and identifying different presentations of reluctance. The interviewers were aware that presentations of interviewee reluctance can vary across individuals, as well as by the same interviewee at different points of the interview (e.g. Henderson et al., 2021). The interviewers gave examples of reluctant presentations, and these included displaying oppositional and challenging behaviours, appearing ‘shut down’ and disengaged, giggling and laughing, appearing jittery and restless, and expressions of heightened emotion (e.g. crying). Regardless of the presentation of reluctance, the interviewers considered a sudden change in behaviour to be a key indicator of reluctance. Thus, they noted that being attentive to the way that interviewees communicate and being present throughout the interview process is essential to identifying reluctance.

I’ve had kids that laugh as they are disclosing. . . . Investigators question that, and say, ‘She seems to think this is funny’, but then the adolescent explains why they’re like that by saying, ‘I’m sorry if I’m laughing or smiling, I’m just really uncomfortable talking about this’. (P09, master’s degree, 6 years’ experience)

I’m always looking out for a change of affect, no matter what that might look like. It could be a kid who’s really talkative and then suddenly shuts down or looks down at the ground, or all of a sudden, they start to get really jittery. (P10, master’s degree, 9 years’ experience)

Two key strategies were recommended in connection to recognising and identifying reluctance: pre-interview preparation and narrative practice.

##### Pre-interview preparation

The interviewers reflected that the information shared during pre-interview meetings with key stakeholders (e.g. the case investigator) often provides their first insight into the interviewee’s readiness to share their experiences, and these meetings help them to prepare for potential reluctance. Examples of information that the interviewers considered helpful included the nature of the disclosure, such as whether the initial disclosure was intentional or accidental, the degree of support received in response to the interviewees’ initial disclosure, and whether the interviewee is aware of the police report. The interviewers noted that personal information about the interviewee also helps to identify reluctance, such as how the interviewee generally presents when they are distressed or disengaged, as well as previous and current mental health diagnoses (e.g. depression, anxiety). Background information was also considered useful because it provides insight into potentially helpful support strategies for that interviewee, such as information about support strategies their caregivers had used previously. Knowledge about the interviewee and their disclosure ultimately helped the interviewers to better understand the context of the interviewee and prepare for possible barriers to disclosure.

There are sometimes concerns that the adolescent could recant their initial disclosure because a parent, or whoever they’ve disclosed to, hasn’t been supportive. So, if the child has taken some heat for their initial disclosure, that indicates to me that it might be really challenging for them to open up again and talk about it in the interview. (P04, master’s degree, 14 years’ experience)

I ask parents what their kid looks like when they are struggling or what has worked to engage them in the past. They know their kid best, so hearing from them is always helpful. I might ask them ‘How do you know that your kid is struggling? What does that look like?’. (P03, master’s degree, 9 years’ experience)

##### Narrative practice

Narrative practice was perceived as an opportunity to build rapport and facilitate the identification of reluctance. In line with best-practice interviewing recommendations (e.g. Roberts et al., 2011), the interviewers indicated that narrative practice is a key rapport-building strategy that can ameliorate the likelihood that reluctance will arise at a later point in the interview. The interviewers noted that should interviewees express reluctance at a later point in the interview, narrative practice helps to establish the rapport necessary to explore that reluctance together, in part because it provides an opportunity for the interviewer to demonstrate attentiveness and genuine interest in what the interviewee has to share. Beyond these benefits, the interviewers perceived that narrative practice could shape a baseline understanding of the interviewee’s presentation and communication, against which any changes in behaviour can be assessed. This baseline understanding of how interviewees communicate was also considered to be helpful, because in some cases, it can be difficult to distinguish reluctance from the interviewee’s natural way of communicating.

Part of rapport building with adolescents is learning their language and how they communicate. Are they communicating in two-word sentences or is this a kid that really likes to chat and can give me a narrative? (P20, bachelor’s degree, 30 years’ experience)

I’m just letting them talk about their life and I’m not condemning them. They just get to talk without judgement, for example about who their friends are, so they get more comfortable with me in turn. (P06, bachelor’s degree, 6 years’ experience)

#### Explore reluctance

The interviewers explored reluctance with the interviewee when it did arise. Exploring reluctance was considered beneficial because it was inherently supportive and could also elicit useful evidential information. Indeed, the interviewers reflected that providing interviewees with the space to explore their perspective and concerns towards disclosure with an attentive interaction partner could help them feel more comfortable to share their experiences. The interviewers perceived that for some interviewees, exploring concerns in this way confers sufficient support in itself, and there is no additional need to address or resolve the interviewee’s concerns.

When further support strategies are necessary, the interviewers noted that exploring reluctance could help inform which support strategies would be most appropriate. In line with research, the interviewers were aware of the myriad of reasons behind why interviewees may present as reluctant, such as their relationship to perpetrator and fear of the consequences of disclosure (e.g. Alaggia et al., 2019). The nature of responses indicated that, in a given moment of reluctance, it is often unclear which concerns are uniquely affecting that interviewee. Thus, the interviewers perceived that exploring reluctance is important to facilitate their understanding of interviewee’s perspective and to enable them to respond appropriately.

In addition to being supportive, the interviewers considered exploring reluctance to go hand in hand with effective evidence gathering. For instance, they perceived that exploring concerns enhances the likelihood of disclosure: the interviewers noted that it is easier to facilitate disclosure when there is some degree of engagement from the interviewee, and exploring reluctance often helps to stimulate dialogue. Indeed, the interviewers gave several examples of when an exploration of reluctance led to disclosure.

The interviewers also described how exploring reluctance can help to elicit useful evidential information, even when interviewees ultimately decide not to disclose. For instance, understanding concerns about the consequences of disclosure can elicit information about the presence of threats and harassment. In cases wherein interviewees perceive the relationship with adult offender as mutual and consensual, exploring the interviewees’ perspective can elicit information about the nature of the relationship and interaction, including potential grooming, and other corroborating evidence such as locations visited, and other activities (e.g. dates). The interviewers additionally noted that exploring reluctance can yield information that contextualises the context of the interviewee and their allegation (or non-disclosure), as well as their presentation in the interview (e.g. shifts in behaviour, or challenging behaviours that evaluators could misinterpret). These factors can ultimately support the credibility of the allegation and the interviewee should the video-recorded interview be used as evidence-in-chief at trial.

By exploring reluctance, you can learn a lot more evidentiary information. For example, nudes that we didn’t know about beforehand. I also think that exploring reluctance is part of the full story. We could learn that an adolescent was scared to tell their mum about what happened because the offender told them they would circulate nude photographs of them if they told the police. All of that information is part of the big puzzle, and if you’re missing some of those puzzle pieces, a jury will wonder why, for example, she didn’t just tell her mom? (P03, master’s degree, 9 years’ experience)

##### Strategies to explore reluctance.

The interviewers explored reluctance through three primary strategies: explore feelings, explore thoughts and explore topics that the interviewee is willing to share. These are described below with accompanying examples. Irrespective of the specific approach used, the interviewers stated that it is often important to continue to explore reluctance beyond initially inviting the interviewee to share their concerns. The interviewers described how exploring reluctance through the application of best-practice questioning skills is often essential to attain insight into their perspective and, at times, could naturally lead into disclosure.

I always want to explore more, whatever the child says, I’ll respond with, ‘Tell me more about XYZ’. If the child says ‘I’m worried about getting into trouble’, I’ll ask ‘Tell me more about getting into trouble’, because I want to understand where this concern is coming from, has someone spoken to this child about this, is it something that the child is hearing from friends. By asking an open-ended question, I’m trying to get all that information. I’m not jumping straight into providing reassurance because I don’t know what is going on for that child yet. It’s always about digging more and asking more questions. (P11, master’s degree, 6 years’ experience)

###### Explore feelings

Interviewers stated that when interviewees initially present as reluctant to talk, they explore the interviewee’s feelings towards the interview, rather than what they were experiencing during the experience that precipitated the interview itself.

I might ask up front ‘how do you feel about being here today?’ instead of explaining the purpose of the interview to them. If they’re coming in and I can tell that they are pissed off, I want to hear that right away, so that I can try to breakdown that barrier with them. (P03, master’s degree, 9 years’ experience)

If they seem to be shutting down, for example when we’re in the middle of the interview and I get the sense that there are things that they are not ready to tell me or they don’t feel comfortable to talk about it, I generally ask ‘How are you feeling right now?’. (P10, master’s degree, 9 years’ experience)

###### Explore thoughts

In some cases, the interviewers perceived that interviewees find it difficult to express their feelings. When this is the case, they considered it helpful to inquire more generally about the interviewee’s thoughts in a given moment. They noted that this strategy often helps interviewees to vocalise their perspective on the interview and the reasons underlying their reluctance. The interviewers stated that in some situations, it is appropriate to specifically inquire about the interviewee’s concerns towards the interview and/or disclosure, as this approach often elicits information about specific psychological barriers to disclosure.

If they are reluctant, for example, they’re not talking and shutting down, or there is that change of affect, I’ll say ‘What’s going on – tell me your thoughts?’ or ‘Tell me what you’re thinking about right now?’. I’m inviting the interviewee to tell me instead of making a judgment based on their appearance. I’m inviting them to tell me what’s going through their mind. (P09, master’s degree, 6 years’ experience)

If I’ve already asked the child how they are feeling about being there, and I get a disengaged response, I might shift to asking more specifically about worries, ‘What are your worries about being here right now?’. (P11, master’s degree, 6 years’ experience)

###### Explore topics that the interviewee is willing to share

Finally, some interviewers noted that they explore topics that the interviewee is willing or unwilling to share, and they also explore the reasons why these topics are or are not okay to discuss in the interview. When explaining this approach, the interviewers noted that even when interviewees are unwilling to disclose, there are often related topics that they are comfortable discussing, and exploring these topics can facilitate dialogue and potentially useful evidential information.

For a lot of the resistant kids, there may be a particular aspect of their experience that they are really resistant to talking about. But in general, they’ll talk about some aspects of it, and so then what we do is go with whatever pieces they are willing to talk about. They may not want to talk about the sex acts, but talk about how they met their boyfriend, or what their boyfriend means to them, and that’s also really relevant to the investigation. (P19, master’s degree, 20 years’ experience)

#### Respond to reluctance

The interviewers commented that in some cases, there is a need to respond by directly addressing or accommodating reluctance. Prior to addressing or accommodating reluctance, however, the interviewers emphasised that it was first important to validate the perspective of the interviewee. The interviewers did so in a variety of ways, including appreciating when the interviewee shared thoughts and feelings (e.g. ‘Thank you for explaining to me how you feel. It is important for me to understand what you think and what you feel about all of this’), normalising feelings or concerns (e.g. ‘That makes sense that you’re concerned about that’) and acknowledging sharing efforts (e.g. ‘I see this is a hard question for you’).

##### Addressing reluctance

At times, the interviewers addressed reluctance by providing information directly relevant to the interviewee’s concerns or another barrier. The interviewers addressed reluctance in one of two ways depending on the context of the interviewee. In some situations, they noted that concerns can be reasonably addressed by enhancing the transparency of the interview process and sharing information. That is, when interviewees have concerns about the interview process (e.g. When they asked who is watching the interview), the interviewers recommended providing information that directly addresses their concerns. In other situations, they considered that reassurance could be provided through supportive statements that helped to ameliorate barriers to disclosure. The interviewers noted that reassurance could be provided on a variety of topics. For instance, when interviewees appear to be withholding information to retain control, the interviewers provided reassurance through statements promoting *autonomy* or *privacy* in other contexts, such as when interviewees appear to be struggling with overwhelming emotions, the interviewers provided reassurance about *pacing*, *feelings* or *normalising the conversation.* Examples of associated supportive statements for these forms of reassurance are included in Table 2.

##### Accommodating reluctance.

In some situations where further support strategies are necessary, the interviewers did not consider it appropriate to address reluctance because the interviewer could not provide honest information that could dispel concerns. In these situations, the interviewers noted that they often accommodate reluctance – an approach that gives the interviewee space not to verbally report their experience (or aspects of that experience), whilst enabling information-gathering to continue. This technique was often (but not always) described as particularly appropriate when interviewees were emotionally overwhelmed at a certain point in the interview, a form of indirect reluctance (Lewy et al., 2015).

###### Re-direct line of questioning

One strategy the interviewers used to accommodate reluctance was to redirect the line of questioning. Specifically, the interviewers took a step back in their approach and asked about peripheral information connected to the interviewees’ experience, rather than continuing to probe about the central aspects of the narrative. The peripheral information that the interviewers inquired about included early complaints, the setting of the experience, what happened at the beginning of the encounter and temporarily re-directing the line of questioning to a neutral topic (e.g. topics from narrative practice).

It’s a lot of just monitoring about how far they are willing to go and seeing what pieces they will engage on. I’m getting them to talk around things and then we might circle back around to the tougher stuff. Only later in the interview, after they’ve had more of a chance to sniff and smell the interviewer, to sniff and smell me, and feel how I respond, will I attempt to get back into something that is more detail oriented about the sexual abuse or sexual assault. (P19, master’s degree, 20 years’ experience)

###### Offer the opportunity to write it down

Another way that the interviewers accommodated reluctance was by giving interviewees the option to write down their narrative, or parts of their narrative. The interviewers considered that some adolescents find it difficult to vocalise sexually elicit details, and in these situations, the process of writing down their experience and having the interviewer read their script out loud, can help to both desensitise the language used in the interview and normalise the conversation.

If they are having trouble verbalising it, it’s just too embarrassing or there’s some kind of shame behind it, I will ask them if they would like to write it down. I think it’s a pretty good block removal technique because when I come back in the room, I'll usually read what they’ve written out loud, and invariably it’s still not detailed enough, but that exercise of writing it down usually breaks that barrier down. Then I'm allowed to ask questions to fill in those details and fill in those blanks. (P16, master’s degree, 21 years’ experience)

## Discussion

Despite consensus on the need to provide reluctant interviewees with socioemotional support, how to address interviewee reluctance on a practical level that goes beyond recommendations to employ a supportive statement is unclear. There is need for a nuanced and practical guide on how interviewers can navigate conversations when interviewees are reluctant. In the current research, we sought to provide insight into this question by exploring how expert interviewers approached and navigated interviewee reluctance. The overriding finding was that interviewers’ response was based on leaning into and exploring the interviewee’s perspective, and tailoring their response based on an awareness of the unique needs of the interviewee. In the following section, we consider this approach in more detail, and discuss the implications of these findings for practitioners and future research.

### An exploration of reluctance

Reluctance was conceptualised as an opportunity to gain a deeper understanding of the interviewee as a person and their whole experience. This perspective is more nuanced than that evidenced in the current literature wherein supportive practices are recommended as a tool to motivate children to discuss their experiences of abuse. Indeed, research on supportive strategies often explore the provision of support strategies and children’s disclosures (Ahern et al., 2014, 2018; Hershkowitz & Lamb, 2020; Saywitz et al., 2019) and the productivity and/or accuracy of their responses (Blasbalg et al., 2018; Hershkowitz et al., 2015; Lewy et al., 2015; Saywitz et al., 2015; Teoh & Lamb, 2013). Evidence indicates that support strategies (typically those included in the revised NICHD protocol) are an effective way to create an environment that helps children to feel comfortable to disclose and share information (e.g. Blasbalg et al., 2018; Hershkowitz et al., 2015; Lewy et al., 2015). Yet even when support strategies are offered, some interviewees will not disclose (Ahern et al., 2019). In these cases, the expert interviewers’ perceived reluctance as an opportunity to understand the interviewee and their experience, rather than a problem or obstacle that needed to be overcome.

The benefits of leaning into reluctance appear to be two-fold. First, it may result in the elicitation of valuable information about the case and interviewee, even in the absence of disclosure. In this study, the expert interviewers considered an exploration of reluctance to be informative in and of itself because it could facilitate the elicitation of additional evidentiary information and bolster the complainant’s credibility. Indeed, an exclusive focus on overcoming reluctance to gather forensically relevant detail may risk a failure to gather information relevant to understanding the interviewee’s *whole* experience, resulting in a narrow account of events, which could limit the information gathered and the credibility of the account attained (Hodgson et al., 2020; Tidmarsh et al., 2012).

Second, exploring reluctance may help children to feel more comfortable to share information. The idea of leaning into reluctance is consistent with the notion of *rolling with resistance,* a foundational principle in the Motivational Interview (Moyers & Rollnick, 2002; Surmon-Böhr et al., 2020). The Motivational Interview has a strong background of empirical support as an approach efficacious in exploring and resolving ambivalence about a diverse range of behaviours, including health-related behaviours such as smoking cessation and improving eating patterns (Lundahl et al., 2010; Magill et al., 2018), as well as facilitating engagement in interviews with high-value detainees (i.e. terrorist suspects; Surmon-Böhr et al., 2020). A foundational principle in the Motivational Interview is ‘rolling with resistance’, which involves upholding interviewees’ autonomy and freedom of choice in order to navigate feelings of ambivalence (Miller & Rollnick, 2002). Ambivalence towards change that is recognised in the Motivational Interviewing approach is comparable to the known experiences of victims of abuse, who typically feel ambivalent towards disclosure, weighing up the benefits of sharing information against perceived negative consequences (McElvaney & Culhane, 2017; McGill & McElvaney, 2023). Exploring reluctance to resolve these feelings of ambivalence may be highly effective in helping interviewees feel more comfortable to share information (Surmon-Böhr et al., 2020). Thus, the tenets of motivational interviewing, especially that of rolling with resistance, may be a helpful grounding framework for future research on responsive support strategies.

### Supporting reluctance requires a foundation of attentiveness throughout the entire interview process

Approaching reluctance stemmed from a foundation of being attentive to the interviewee throughout the entire interview process. Indeed, the interview dynamic was perceived as an ongoing process in which rapport played a key role in resolving reluctance across the interview. Existing literature on the effects of support has been conducted on two primary levels: those that analyse the immediate effect of an interviewers’ response to reluctance on the interviewees informativeness (micro-level analysis; Ahern et al., 2014; Hershkowitz et al., 2006), and those that analyse the aggregated results of a supportive interviewing protocol on interviewee informativeness (macro-level analyses; Ahern et al., 2018; Blasbalg et al., 2019; Hershkowitz et al., 2015; Lewy et al., 2015; Teoh & Lamb, 2013). In addition to these micro- and macro-level analyses, some research also isolates analyses of supportive behaviours and their effect to a single interview phase (i.e. rapport-building or the substantive phase). Our findings suggest that using one of these approaches in isolation limits understanding of the effects of providing nuanced and continuous support throughout the interview. For example, micro-level approaches minimise the role of the relational dynamic between the interviewer and interviewee. Conversely, the aggregated results of macro-level approaches result in limited understanding of the interactional dynamics and provision of support on a practical level.

The finding that supporting reluctance requires attentiveness throughout the entire interview process, which emerged based on perspectives of the expert interviewers in the current study, complements the empirical findings of Blasbalg et al. (2018), who found that in each moment of the interview, interviewee behaviours, particularly reluctance and informativeness, were heavily influenced by the behaviours of the interviewer in the preceding conversational turn. That is, when interviewers responded to interviewee reluctance with a prompt inquiring about the allegation, interviewees’ reluctance often persisted into the next conversational turn (see also Hershkowitz et al., 2006; Katz et al., 2012). Conversely, when interviewers responded to reluctance with support, interviewees tended to be less reluctant, and more informative in response. These empirical results converge with the findings of the current research to underscore the necessity of ongoing interviewer attentiveness, to ensure that reluctance is both recognised and responded to appropriately when it arises.

### A focus on the appropriateness of support in a given moment

Our findings point to the need to provide an appropriate form of support in response to given moments of reluctance. The revised NICHD interviewing protocol (Lamb et al., 2018) provides examples of when various supportive strategies may be useful (e.g. during rapport building, when interviewees demonstrate an overt refusal to engage with the interviewer), alongside example statements. A variety of supportive strategies that were recommended in the current study are consistent with examples that have been included in child-interviewing protocols, including the revised NICHD protocol, such as those that specifically focus on exploring the interviewee’s feelings (Lamb et al., 2018). The current study suggests that interviewers may risk providing ineffective or suboptimal support if they only look at explicit indicators of reluctance in a given moment and rely on a script of supportive statements to inform responses. Rather, understanding the interviewee’s perspective and the reasons for reluctance may be critical for informing interviewers on which pathway is most suitable for addressing or accommodating reluctance.

Further, as reluctance can present in unpredictable forms, it is important that interviewers explore ambiguous behaviour rather than make assumptions about a presentation in each moment (Henderson et al., 2021; Katz et al., 2012). This approach is consistent with the principles of *clinical judgement,* a concept referred to in the medical and psychological literature as informed decision-making when clinicians are faced with ambiguous or complex problems (Tsang et al., 2023). Pillars of effective clinical judgment include knowledge and experience, curiosity, reflection, and attending to contextual factors (Tsang et al., 2023). The expert interviewers’ response to reluctance paralleled these pillars. That is, to select the most appropriate supportive strategies, interviewers approached the situation with a foundation of best-practice interviewing, a curiosity to gather more information about the reluctance and/or the target event, and they considered the interviewee’s behaviour within the context of broader background information (e.g. potential barriers to disclosure). Ultimately, the results of the current study suggest that being well informed about the interviewee and the nature of reluctance positions interviewers to provide the most appropriate forms of support.

### The importance of training in responding to reluctance

Understanding and responding to reluctance should be recognised as an additional skill that relies on a foundation of best-practice interviewing. The investigative interviewers involved in this study were well trained and recognised as highly proficient in using best-practice interviewing skills and were thus able to adopt an adaptive approach based on the needs of the interviewee and the interview. This adaptive approach requires knowledge of the functions of different types of questions (open-ended questions vs. closed-ended questions), the interview structure, the parameters of the interviewer’s role and the underlying purpose of different support strategies (Hershkowitz et al., 2017; Risan et el., 2020; Yi et al., 2016). Hence, the results of this study underscore the importance of high-quality training programmes for investigative interviewers to develop skill in using best-practice questioning (e.g. Brubacher et al., 2022; Cederborg et al., 2021; Hershkowitz et al., 2017). With this foundation in place, the next step is for training programmes to include guidance on how interviewers can navigate reluctance with evidence-based support strategies, a topic that relatively few training programmes have addressed (Hershkowitz et al., 2017). Therefore, it is important that interviewers receive both foundational training in best-practice interviewing and specific training on the use of evidence-based supportive strategies, including how to navigate reluctance.

### Future directions

This study was conducted as a starting point to understand how interviewers can navigate situations with high levels of interviewee reluctance, based on the perspectives of expert interviewers. Thus, strategies that emerged as novel in this study (e.g. exploring reluctance) need to be subject to rigorous empirical evaluation to determine their effectiveness through a more objective lens (e.g. analysis of transcripts, experimental testing; for an example see, Dianiska et al., 2023). Such an evaluation is critical prior to the integration of these methods into recommended practice. Additionally, empirical research on how to effectively train interviewers on navigating reluctance is needed. Although some interviewer training programmes include guidance on interviewer support, guidance is limited to *what* support encompasses (e.g. show interest; Brimbal et al., 2021). Few opportunities exist for interviewers in training to practise *when* or *how* to use support-based skills effectively (Brimbal et al., 2021). Further knowledge is needed on how to design and implement training programmes that effectively foster support-based skills. Finally, interviewees’ experiences of interviewers navigating and supporting reluctance may also help achieve a fuller picture of the effectiveness and impact of various support strategies.

## Conclusion

Many adolescents are reluctant to discuss suspected experiences of abuse when they are interviewed. When this occurs, it is essential that interviewers are adequately equipped to navigate reluctance in a supportive and interviewee-centred way. The current study was conducted to provide insight into how this can be best achieved through the perspectives of expert interviewers. The expert interviewers responded to reluctance by leaning into and exploring the interviewee’s perspective, and tailor their response based on an awareness of the unique needs of the interviewee. These results provide a blueprint for future research to empirically test strategies that were recommended in the current research, though these strategies should not be considered in isolation from the necessity of effective and ongoing rapport. Empirically testing support strategies is paramount to enable the inclusion of evidence-based support strategies in best-practice interviewing protocols, so that investigative interviewers are prepared to respond to reluctance when it does arise.
